# A significant correlation exists between *CREBBP* and *CEBPA* gene expression in *de Novo* adult acute myeloid leukemia

**DOI:** 10.1038/s41598-025-93024-2

**Published:** 2025-04-11

**Authors:** Magda Assem, Asmaa A. El Leithy, Naglaa M. Hassan, Ahmed A. Al-Karmalawy, Mohamed Abozaid, Rasha Mahmoud Allam, Mohamed A. M. Kamal, Marwa Amer, Gharieb S. El-Sayyad, Noha H. Ibrahim

**Affiliations:** 1 Clinical Pathology Department, National Cancer Institute, Cairo University, Giza, Egypt; 2 College of Biotechnology, Misr University for Science and Technology, Giza, Egypt; 3 Department of Pharmaceutical Chemistry, College of Pharmacy, The University of Mashreq, Baghdad, 10023 Iraq; 4 Department of Pharmaceutical Chemistry, Faculty of Pharmacy, Horus University-Egypt, New Damietta, 34518 Egypt; 5 Hamilton Lab, School of Medicine, Dentistry and Nursing, Anderson College, University of Glasgow, Glasgow, Scotland, UK; 6 Department of Cancer Epidemiology and Biostatistics, National Cancer Institute, Cairo University, Giza, Egypt; 7 Clinical Pathology Department, El-Hussein University Hospital, Al-Azhar University, Cairo, Egypt; 8 Bioinformatics and Functional Genomics Department, College of Biotechnology, Misr University for Science and Technology (MUST), Giza, Egypt; 9 Medical Laboratory Technology Department, Faculty of Applied Health Sciences Technology, Badr University in Cairo (BUC), Badr City, Cairo, Egypt

**Keywords:** *CREBBP*, *CEBPA*, *DNMT3A*, RT-PCR, Acute myeloid leukemia, Diseases, Cancer, Immunological disorders

## Abstract

*CREBBP*, *CEBPA*, and *DNMT3A* are tumor suppressor genes whose dysfunction has been reported in hematologic malignancies. Acute myeloid leukemia (AML) is the most common type of acute leukemia in adults. We aim to assess the expression level of *CREBBP*, *CEBPA*, and *DNMT3A* genes in an Egyptian cohort with AML. We investigated the correlation between the selected genes’ mRNA levels and their association with clinical characteristics and survival. Herein, 53 adult participants diagnosed with AML were enrolled in the study. Quantitative RT-PCR was used, and computational analysis was added to analyze the relationship between the three genes. *CREBBP* expression influenced TLC negatively (*r *= -0.328, *p* = 0.017). *DNMT3A* gene expression was found to be significantly associated with CD117 positive (*p* = 0.028). There was no significant difference between males and females in the relative *CREBBP*, *CEBPA*, and *DNMT3A* expression. Remarkably, AML-M3 cases were devoid of *CREBBP* expression. The correlation matrix of the three genes detected a significant correlation only between *CREBBP* and *CEBPA* expression (*r* = 0.518, *p* < 0.0001), though the computational correlation analysis of these two genes was not significant. Our finding may suggest a complementary role of *CREBBP* and *CEBPA* in AML pathogenesis; however, further investigation on larger samples is still warranted to study the relationship of these genes with AML survival. We are also reporting here an adult AML case with an additional chromosome 19 as the sole cytogenetic abnormality.

## Introduction

Acute myeloid leukemia (AML) is a very heterogenous disease in adults^1^. It occurs when the accumulation of clonal myeloid precursors encroaches upon normal bone marrow elements with consequent impairment of normal erythropoiesis and granulopoiesis. Genetic or epigenetic alteration of hematopoietic stem cells remains the direct cause^2^. Chromosomal translocations, inversions, and gene mutations were reported in some patients. Among them, t(8; 21)(q22q22.1), inv(16)(p13.1q22), and mutations in *NPM1*,* FLT3*,* RUNX1*, and *TP53* genes. What they all have in common is altering the differentiation of myeloid stem cells in their early stages of development^1^.

Cyclic-AMP response element binding protein (CREBBP) is an ubiquitously expressed gene that plays a significant role in hematopoiesis^3,4^. It acts as a scaffold coactivator for many transcription factors and has intrinsic acetyltransferase activity. Absent CREBBP activity fails histone acetylation and RNA polymerase II recruitment. CREBBP maintains the balance between hematopoietic stem cell lineages, as evidenced by the biased differentiation toward myeloid cells and decreased stem cell population after its ablation in adult mice^5^.

Chromosomal aberrations disrupt *CREBBP* on chromosome 16p13, such as inv(16)(p13.1q22) or t(8;16)(p11;p13), usually resulting in AML with distinct morphological and clinical features^6–8^. Recently in the fifth edition of the World Health Organization (WHO) classification of Tumors, the fusion of *KAT6A*::*CREBBP* t(8;16) (p11.2;p13.3) was included under the category AML with other defined genetic alterations. Also, *CREBBP* was included as one of the many partner genes reported in *KMT2A* rearranged AML^9^. Despite this clear association of *CREBBP* with AML, its prognostic role and interaction with other genes in this disease’s pathogenesis need to be elucidated.

CCAAT/enhancer-binding protein alpha (*CEBPA*) is a single-exon gene found on chromosome 19q13.1 that encodes for an essential leucine zipper protein^10–13^. It initiates the expression of specific genes required to differentiate myeloid progenitor cells by binding to the DNA motif, CCAAT, to enhance the transcription. While the association of *CEBPA* mutations with AML outcome is well known^14–17^, the impact of its expression level on subtype and survival is inconsistent among studies^18–20^.

DNA methyltransferase 3 alpha *(DNMT3A)* is a 23-exon gene mapped to chromosome 2p23, which encodes for a protein responsible for methylation of cytosine residue at the CpG islands, therefore, represses gene transcription^21,22^. *DNMT3A* mutation and overexpression were reported in AML cases^23–25^. The exact link to how *DNMT3A* mutation or overexpression predisposes to AML is unknown^22^. Therefore, correlating *DNMT3A* expression level with other genes and clarifying its clinical significance was included in this study.

Many studies reported a relationship between the mutation statuses of *CEBPA* and *DNMT3A* in determining the prognosis and treatment outcome of AML^9,12,22,25,26^; however, the literature correlating their mRNA expression is limited. In a Japanese study (including 605 patients) in 2023 *DNMT3A* ^R882^ mutation was found to be an independent factor for poor prognosis^27^. Among 123 Egyptian adult AML patients *DNMT3A* mutation affected complete remission (CR) negatively and in combination with *FLT3* mutation had a significant lower overall survival (OS) rate^28^. In 2024 among 1010 *CEBPA*-mutant adult AML patients detailed mutational analysis (bZIP^InDel^, bZIP^STOP^, bZIP^ms^, TAD, and sm*CEBPA* vs. dm*CEBPA* mutations) revealed only patients with bZIP^InDel^ mutation had significatly higher CR and OS^29^. Similarly, an interaction may exist between *CEBPA* and *CREBBP* functions^30^, but to our knowledge, it has never been investigated in leukemia. Understanding the genetics of AML pathophysiology is crucial to design targeted therapies, determine the prognosis, and improve survival.

Therefore, we aim to assess whether the expression level of the selected genes differed regarding the clinical and pathological features of AML or had any impact on either overall or disease-free survival. Furthermore, we analyzed the correlation between their expression levels to examine their interrelationship.

## Materials and methods

### Study group

53 participants were newly diagnosed with AML at the Hematology Clinic, National Cancer Institute. The diagnosis was made according to French-American–British (FAB) Cooperative Group Criteria. Written informed consent was obtained from all participants. The protocol was approved by the Institutional Review Board (IRB) of the National Cancer Institute (NCI), Cairo University, Egypt, following the principles of the Declaration of Helsinki (IRP Approval No. CP2403-303-022). All patients underwent thorough history-taking, clinical examination, and routine laboratory tests: complete blood count (CBC), bone marrow (BM) aspiration and morphology, flow cytometry, and chromosome and gene tests (mutations in *FLT3* gene and conventional karyotyping). Patients with acute promyelocytic leukemia (APL) were offered All-trans retinoic acid (ATRA). Other FAB subtypes were given the 3 + 7 treatment protocol: three days of daunorubicin, at least 60 mg/m^2^, idarubicin, 10–12 mg/m^2^, or the anthracene-dione mitoxantrone, 10–12 mg/m^2^, followed by 7 days of cytarabine (100–200 mg/m^2^ continuous IV). Response to induction therapy was assessed between days 14 and 28 after induction therapy. Fifteen PB samples from matched age and sex healthy donors from the same hospital enrolled in this study as a control group. A group of publicly available gene expression data for this study targeted genes from 173 AML patients from the cancer genome atlas (TCGA) data, which was selected for comparison to validate this study’s findings.

#### RNA isolation and cDNA synthesis

Per the manufacturer’s instructions, total cellular RNA was isolated from peripheral blood (PB) samples obtained during routine laboratory tests using the QIAamp RNA Blood Mini Kit (QIAGEN, Hilden, Germany). RNase-free DNase (QIAGEN, Hilden, Germany) was used to remove any contaminating DNA according to the manufacturer’s instruction, and then concentration was measured by NanoDrop (Thermo Fisher Scientific). After that, cDNA synthesis was completed using QuantiTect Reverse transcription kit (QIAGEN, Hilden, Germany) according to the manufacturer’s instruction using RNA 0.02 µg/µl from each sample on PCR Thermal Cycler (Applied Biosystems, Life Technologies, USA).

#### Quantitative real-time PCR (qRT -PCR)

Quantitative analysis of *CEPBA*,* CREBBP*, and *DNMT3A* expression, as well as the reference gene Glyceraldehyde 3-phosphate dehydrogenase (*GAPDH*), was performed using C1000 Touch™ Thermal Cycler according to the manufacturer’s protocol for QuantiTect SYBR^®^ Green PCR Kits (Qiagen, Hilden, Germany). Primers for target and housekeeping genes were designed using GenBank https://www.ncbi.nlm.nih.gov/genbank/, and the sequences are provided in Table 1. The final volume of the two-step PCR reactions was in 50 µl containing cDNA (100 ng), 25 µl of 2X QuantiTect SYBR Green PCR Master Mix, 0.5 of uracil-N-glycosylase, and RNase-free water. The cycling conditions were as follows: UNG carryover prevention for 2 min at 50 °C, PCR initial activation for 15 min at 95 °C, denaturation for 1 min at 95 °C, annealing for 30 s at 55 °C, elongation at 72 °C for 30 s then repeated for 35–40 cycles. Negative controls without a cDNA template were considered for each experiment. Firstly, the *GAPDH* gene expression was tested as a study reference gene, and an expression value was detected in all samples. All samples were run in triplicate. The expression levels are relative to *GAPDH* in each patient as an internal control. Cycle threshold (CT) values were collected and normalized to *GAPDH*. All genes’ relative expression (fold changes) was calculated using the 2^−ΔΔCT^ method compared to normal individuals. A cut-off value of the fold change median for each gene was considered. Data at the median value and between the median and below it, the gene was considered downregulated. The gene was considered upregulated above the median gene expression fold change value.

**Table 1:** Forward and reverse primers for each of the *CEPBA*, *CREBBP*, *DNMT3A*, and the reference *GAPDH* gene as designed from the GenBank (https://www.ncbi.nlm.nih.gov/genbank/).

Gene name	Forward primer 5′–3′	Reverse primers 5′–3′
*GAPDH*	CTCATGACCACAGTCCATGC	TTCAGCTCTGGGATGACCTT
*CREBBP*	GACGACCCTTCACAGCCCCAG	TTCAAGCAGTTGTCGCACAC
*CEBPA*	TCGCCATGCCGGGAGAACTCTAAC	CTGGTAAGGGAAGAGGCCGGCCAG
*DNMT3A*	GTGTGGTTAGACGGCTTCCG	CCCATGTCCCTTACACACACG

#### Immunophenotyping

A panel of monoclonal antibodies targeting myeloid-associated antigens, including CD4, CD7, CD14, CD11c, CD117, HLA-DR, and CD34 was used to characterize the phenotypes of the leukemia cell with Epics XL4 flow cytometer (Beckman Coulter, USA).

#### Data acquisition and preprocessing

The mRNA expression dataset utilized in this study was procured from the cBioPortal for Cancer Genomics (TCGA-LAML). We specifically selected the “mRNA Expression, RSEM (Batch normalized from Illumina HiSeq_RNASeqV2)” for 173 AML patients (median age 58 years ranged from 18 to 88 years) dataset from the Acute Myeloid Leukemia (TCGA, PanCancer Atlas) project. This dataset encompasses a comprehensive collection of gene expression profiles from various samples, providing an extensive overview of mRNA expression patterns in AML.

### Statistical analysis

Statistical analysis was conducted using SPSS V.25 (IBM Corporation, Chicago, IL). Numerical data were expressed as mean and standard deviation or median and range according to their distributions. Differences in the expression levels were examined using the non-parametric tests, Mann-Whitney U and Kruskal–Wallis, concerning the qualitative variables and using Spearman’s rank correlation coefficient relating to the quantitative ones. Categorical associations were examined using Chi-square and Fisher’s Exact tests as appropriate. Survival analysis was estimated using the Kaplan–Meier method, and then the log-rank test assessed further Analysis of univariate statistical significance. Statistical significance was set at less than 0.05 for all analyses. For the data retrieved from the TCGA, we performed a pairwise correlation regression analysis to examine the relationships between the expression levels of the selected genes. Pearson’s correlation coefficient was employed to assess the strength and direction of the linear relationships between gene pairs. A regression analysis was conducted for each pair to further elucidate their linear association. The significance of these correlations was determined through *p*-value calculations.

## Results

The study was conducted on 53 patients (27 females and 26 males) with a mean age at the time of diagnosis of 40 ± 1.9 years. Patients’ characteristics and clinical laboratory findings are presented in Table 2. The profiling of *CREBBP*, *CEBPA*, and *DNMT3A* in PB cells of AML patients showed differential expression compared with their expression in the control group. Although the survival curves are somewhat separated through survival analysis, the results showed no statistically significant association between the studied patient’s OS or DFS and the gene’s expression levels. Based on that, the patients with M3 and non-M3 AML were analyzed together to avoid the limited numbers within strata. The fold changes of the studied genes were then analyzed to define lower and higher expression groups, as shown later in the results.

**Table 2:** Clinical-laboratory data of the studied group (*n* = 53).

Characteristics	Number of patients (*n* = 53)%	
Sex	53	
Male	26	49.1
Female	27	50.9
Age at AML diagnosis (years) Mean (SD)	40 (1.9)	
*FLT3-ITD*	49	
W/W	41	83.7
W/ITD	8	16.3
Leukemia subtype according to FAB classification	51	
M1	13	25.5
M2	10	19.6
M3	13	25.5
M4 or more	15	29.4
Missed	2	
Cytogenetics	37	
Normal	22	62.2
t(8;21)	1	35.1
Abnormal + ve for PML-RARA fusion gene or t(15;17)	13	2.7
47, xy, + 19	1	
Total leukocytic count (TLC) (*10^9^/L) median (range)	22 (0.5-266.32)	
Platelet (PLT) (*10^9^/L) median (range)	26 (3-137)	
Hemoglobin (Hb) (g/L) median (range)	7.7 (2.3–10.3)	
Initial BM blast median (Range)	54% (30–98%)	
Initial BM promyelocyte median (range)	9.4% (5-98%)	
Mortality	49	
Alive	28	57.1
Dead	21	42.9
Lost follow-up	4	

### Relative expression level of *CREBBP*, *CEBPA*, and *DNMT3A* in the study group

*CEBPA* and *DNMT3A* expression were upregulated in 47 (88.7%) and 51 (96.2%) out of 53 participants. *CREBBP* was upregulated in only ten participants (19%) out of our all samples. The expression level of the *CEBPA* gene ranged between 0.001 and 7.763 and had a median value of 0.4555, while it was between 0.0031 and 12.8836 with a median value of 0.1346 for the *DNMT3A*. On the other hand, the *CREBBP* relative expression median value was 0.0112, with minimum and maximum values of 0.003 and 0.0928, respectively.

### *CREBBP*, *CEBPA*, and *DNMT3A* expression level and patient’s clinicopathological data

The expression of *CREBBP*,* CEBPA*, and *DNMT3A* was compared with all clinical characteristics of each patient. None of the genes’ expression levels showed a statistically significant difference between males and females, *p*-value = 0.175, 0.364, and 0.388, for the three genes, respectively. The mean age at the time of AML diagnosis was 40 ± 1.9 years, and no significant correlation was detected between age at the time of diagnosis and either *CREBBP* (*p*-value = 0.322), *CEBPA* (*p*-value = 0.955) or *DNMT3A* (*p*-value = 0.233).

**Fig. 1:**
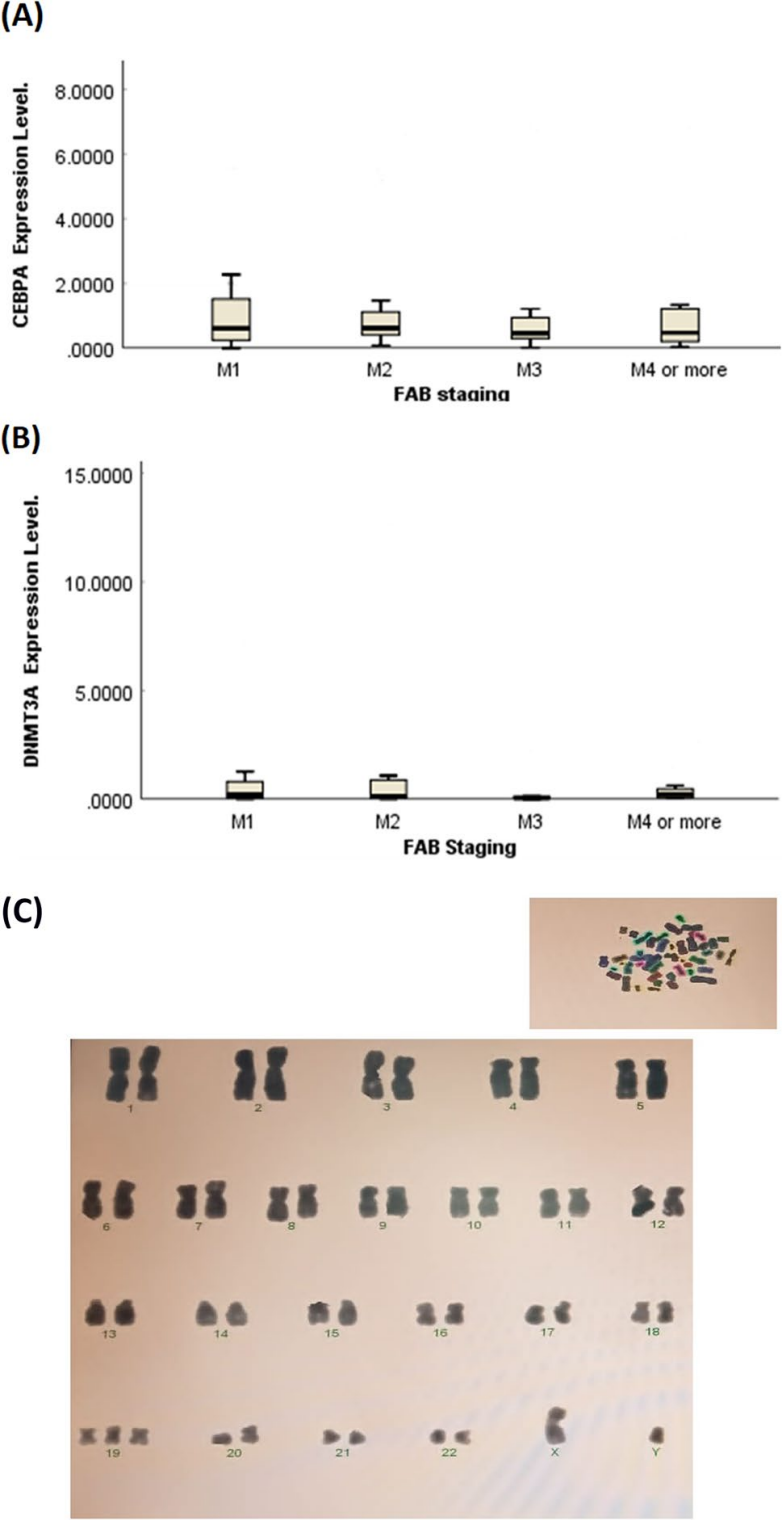
Association between *CEBPA* (**A**) and *DNMT3A* (**B**) genes expression levels and FAB classification. No significant differences were observed (*p*-value = 0.932 and 0.357, respectively). (**C**) Karyotype photo showing additional chromosome 19 as the sole rare cytogenetic abnormality.

In addition, according to the FAB classification of AML, participants were divided into 4 subgroups: M1 (*n* = 13), M2 (*n* = 10), M3 (*n* = 13), and M4 or more (*n* = 15). No statistical differences were found between the relative expression of the studied genes and FAB subtypes, *CREBBP* (*p*-value = 0.087), *CEBPA* (*p*-value = 0.932), and *DNMT3A* (*p*-value = 0.357) (Fig. 1A & B). Notably, 2, 4, 0, and 3 participants had detectable *CREBBP* expression among the four subgroups. Leukemia cell karyotype was available in pateints data for 37/53 (69.81%) participants, 15/37 (40.54%) of which had abnormal karyotypes. Among them, thirteen participants had positive t(15; 17) or abnormal PML-RARA fusion gene,one with t(8;21), and one with 47 XY + 19 karyotype (trisomy 19 shown in Fig. 1C). None of the gene’s expression levels differed significantly between subgroups of patients with abnormal and normal karyotypes.

### Correlation between *CREBBP*, *CEBPA*, and *DNMT3A* expression level with immunophenotypic markers and clinical features

The expression levels of all genes were evaluated according to the presence of HLA-DR, CD117, CD11c, CD34, CD7, CD4, and CD14 surface markers. Analysis revealed no significant differences in mRNA level between any marker positive versus negative participants, except for CD117 positive patients, which showed high expression levels of *DNMT3A*. No correlation was found for *CEBPA* or *DNMT3A* with either Hb level, TLC, or PLT counts; however, a statistically significant negative correlation was detected between *CREBBP* expression level with TLC count (*r*= -0.328, *p* = 0.017), the *p*-value was given in Table 3.

**Table 3:** Association between each selected gene expression with immunophenotypic markers and laboratory findings *n* = 52).

		*n* (52)	*CREBBP* (*p*-value)	*CEBPA* (*p*-value)	*DNMT3A* (*p*-value)
HLA-DR	Present	27	0.729	0.288	0.264
	Absent	25			
CD117	Present	19	0.729	0.549	0.028*
	Absent	33			
CD11c	Present	8	0.642	0.833	0.462
	Absent	44			
CD34	Present	19	0.467	0.305	0.524
	Absent	33			
CD7	Present	5	1.00	0.449	0.526
	Absent	47			
CD4	Present	8	0.642	0.501	0.630
	Absent	44			
CD14	Present	11	0.424	1.00	0.991
	Absent	41			
TLC count	Median (Range) 22 (0.5-266.32)		0.017*	0.340	0.822
Hb level	Median (Range) 7.7 (2.3–10.3)		0.973	0.143	0.258
PLT count	Median (Range) 26 (3-137)		0.306	0.660	0.697
Blast count	Median (Range) 60 (21–97)		0.341	0.670	0.557

*Indicates significance.

### Correlation between *CREBBP*, *CEBPA*, and *DNMT3A* expression level

**Fig. 2:**
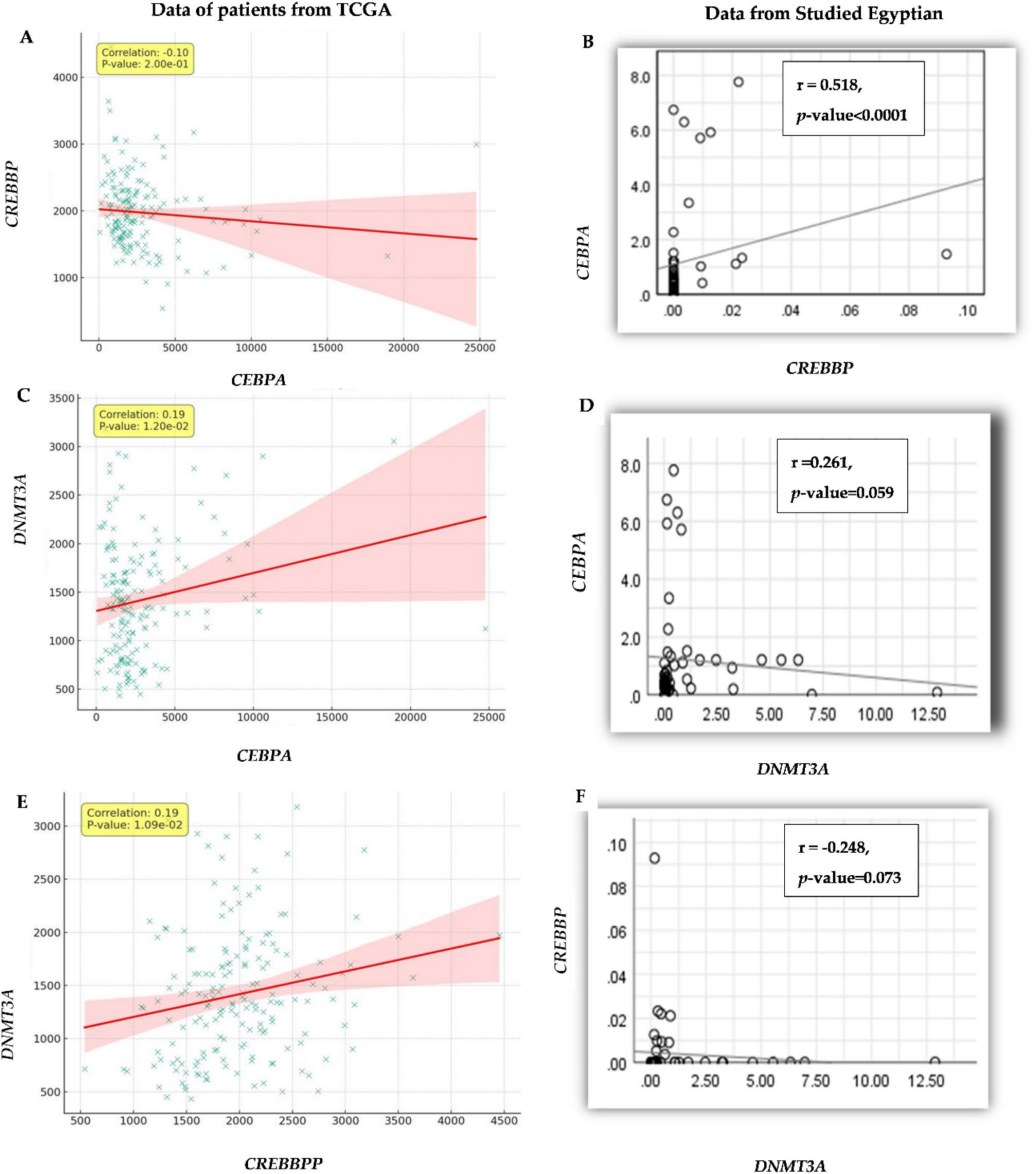
Pairwise Correlation Plots with Regression Lines for Expression data levels of *CREBBP*, *CEBPA*, and *DNMT3A* genes (**A**), (**C**), and (**E**) for the downloaded from TCGA. The scatter plot is annotated with a Pearson correlation coefficient and a *p*-value quantifying the strength and significance of their correlation. (**B**), (**D**), and (**F**) for the current Egyptian cohort.

The correlation between *CREBBP*,* CEBPA*, and *DNMT3A* gene expression levels was examined. We found a highly statistically significant correlation between *CREBBP* and *CEBPA* expression level (r_S_= 0.518, *p-*value < 0.0001), Fig. 2B. We detected a negative trend in the correlation between *CEBPA* and *DNMT3A* despite being statistically insignificant (r_S_=0.261, *p-*value = 0.059), Fig. 2D. No significant correlation was found between expression levels of *CREBBP* and *DNMT3A* (r_S_=0.248, *p-*value = 0.073), Fig. 2F.

Regarding the computational analysis, expression level data of *CREBBP*, *CEBPA*, and *DNMT3A* genes was downloaded from TCGA; analyzing them showed: a) the scatter plot and regression analysis revealed a weak negative correlation not statistically significant (Pearson *r*= -0.0978, *p*-value = 0.2004) between *CEBPA* and *CREBBP* (Fig. 2A). This finding suggests that while there is a slight inverse relationship in their expression levels, it might not be a defining characteristic in the context of AML. Furthermore, the relationship between *CEBPA* and *DNMT3A*, Fig. 2C, was characterized by a statistically significant weak positive correlation with a Pearson coefficient of 0.1907 ( *p*-value = 0.0120). This result may imply a potential regulatory or functional connection between these genes in AML. Lastly, a significant weak positive correlation was observed between *CREBBP* and *DNMT3A*, Fig. 2E, with a Pearson *r* = 0.1930 (*p*-value = 0.0109). This could indicate a meaningful biological interaction between these genes in the pathology of AML.

**Fig. 3:**
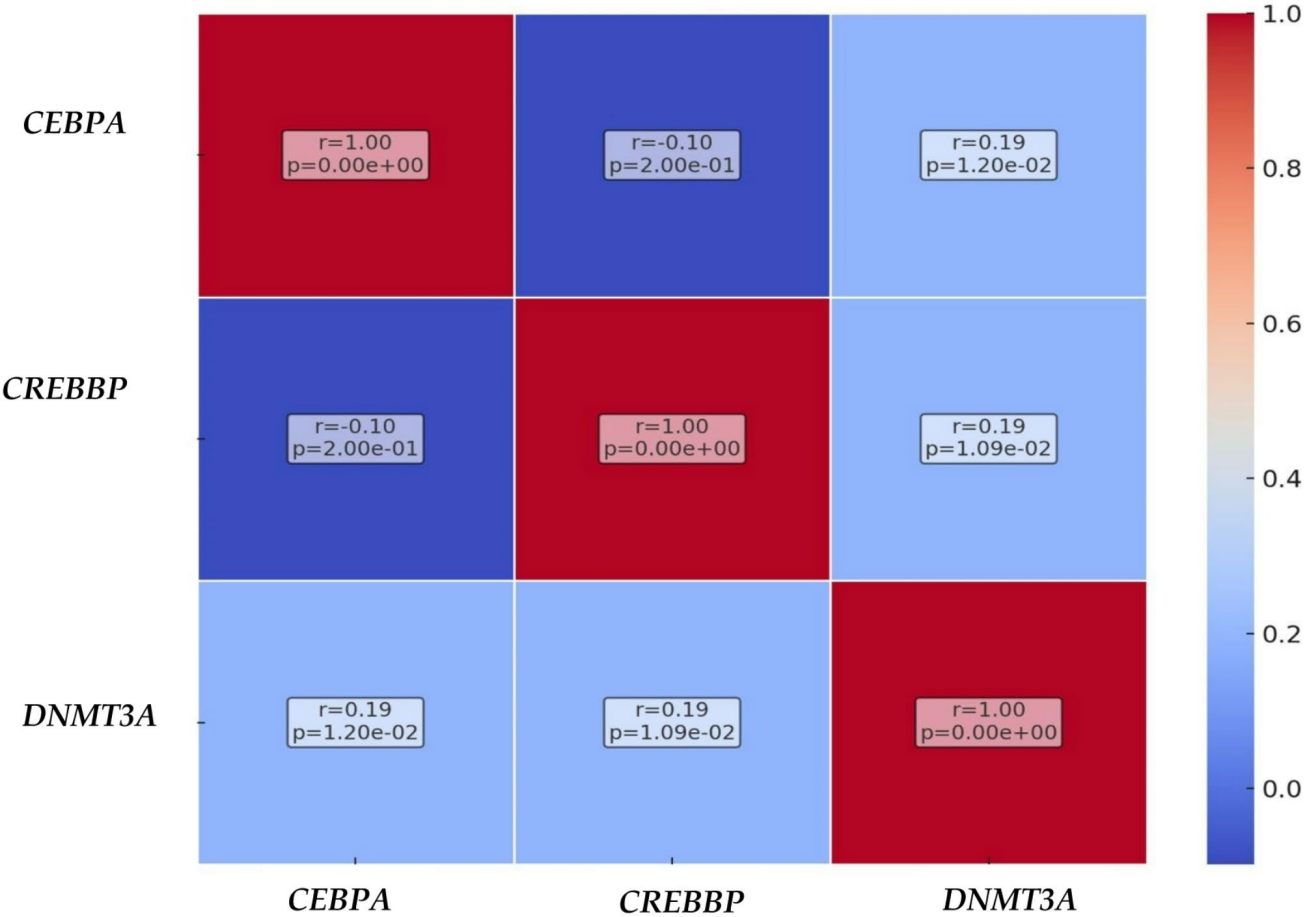
Correlation Heatmap with *p*-values. It features a heatmap that visualizes the correlation coefficients between the pairs of genes: *CEBPA*, *CREBBP*, and *DNMT3A*. Each cell in the heatmap represents the correlation coefficient for a gene pair with the color intensity indicating the strength of the correlation. The cells are annotated with the Pearson correlation coefficient and the corresponding *p*-value.

In addition, the heatmap, Fig. 3, showcases each gene pair’s correlation coefficients and *p*-values, providing a comprehensive view of their interactions. The color intensities in the heatmap reflect the strength of correlations, while the annotations offer immediate insights into their statistical significance. The heatmap highlights the complex network of gene interactions in AML, with some gene pairs showing stronger correlations than others. The statistical significance marked by the *p*-values is particularly crucial, as it underscores the most likely biologically relevant correlations.

### Effect of relative gene expression on response to chemotherapy of the studied patients

**Fig. 4:**
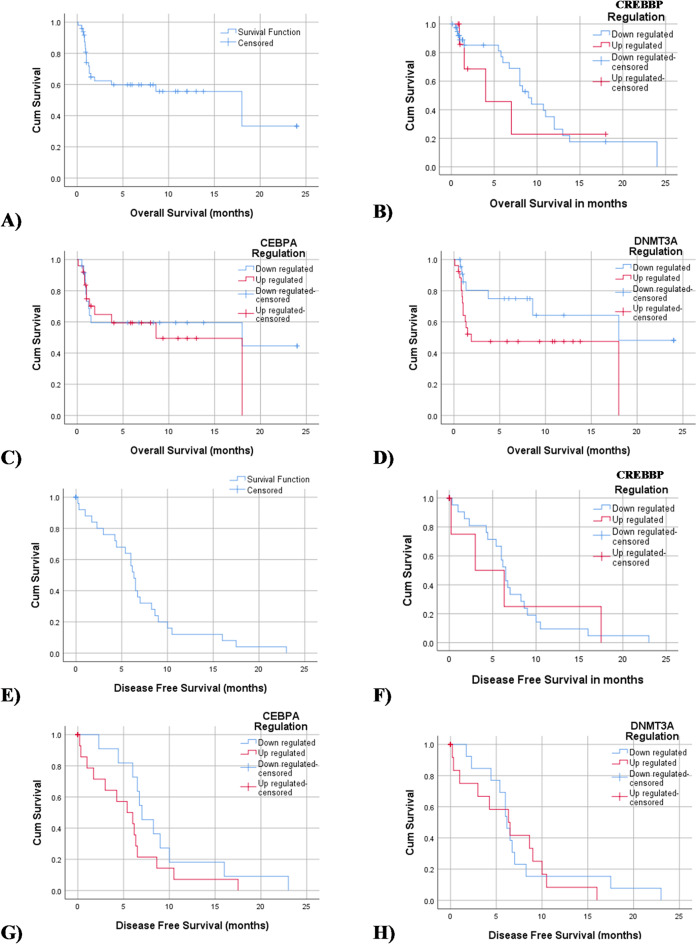
Kaplan- Meier Curves for the Association between each selected gene expression with overall [a-d] and disease-free survival [e-h]. (**A**) Cumulative proportion of overall survival (OS) for all participants. (**E**) Cumulative proportion of disease-free survival (DFS) for all participants. (**B**–**D**) and (**F**–**H**) cumulative proportion of OS and DFS of downregulated vs. upregulated *CREBBP*,* CEBPA*, and *DNTM3A*, respectively.

For the clinical analysis, data on patient response to the treatment was available for 49 (92.45%) patients out of 53 studied patients. Four (7.5%) patients died after diagnosis and before receiving any treatments. Complete remission (CR) was achieved in 47 (95.9%) out of 49 cases who received conventional chemotherapy. Follow-up data were collected for a median duration of 3.75 months (range: 0 to 25 months). The median follow-up time was 3.75 months (0 to 25 months). The overall survival (OS) of AML patients was measured from the date of diagnosis until the date of death or censoring for patients alive at the last follow-up (Fig. 4A). The disease-free survival (DFS) was measured from the date of complete remission till the date of relapse, death, or last follow-up (Fig. 4E).

The association of *CREBBP*, *CEBPA*, and *DNMT3A* expression values with survival was analyzed in the patients who received conventional chemotherapy. These patients were recategorized into 2 groups based on the relative gene expression as up and down-regulated. To avoid the limited numbers within strata, the values of expression above and below the 75th percentile were considered up and down-regulated, respectively. Our analysis showed no statistically significant association between the studied patient’s OS or DFS and the gene’s expression levels (Fig. 4B–D and F–H, respectively) (Table 4).

Moreover, this study evaluated the relationship between patients’ clinical laboratory data and OS and DFS. OS was statistically significant only for the *FLT3*-ITD wild group, which tended to have a longer OS than the mutant one (median survival: 18 months vs. 0.8 months) (*p-* value < 0.001) (Table 5). As for DFS, no clinical laboratory data except *FLT3*-ITD mutation status showed statistical significance. Wild *FLT3*-ITD participants had a longer DFS median of 6.5 months in comparison to mutant *FLT3*-ITD, which revealed a median survival of 0.2 months (*p-*value < 0.0001) (Table 5).

**Table 4:** Association between each of the selected gene expressions with overall and disease-free survival.

Survival type	Gene expression	*n* (49)*	Proportion surviving at 6 months	Median survival estimate (months)	*p*-value
OS	*CREBBP*	Upregulated (9)	0.486	1.9	0.440
		Downregulated (40)	0.898	18	
	*CEBPA*	Upregulated (12)	0.619	NR	0.595
		Downregulated (37)	0.889	18	
	*DNMT3A*	Upregulated (11)	0.636	18	0.828
		Downregulated (38)	0.919	18	
DFS	*CREBBP*	Upregulated (9)	0.500	3	0.918
		Downregulated (38)	0.571	6.5	
	*CEBPA*	Upregulated (12)	0.857	6.3	0.468
		Downregulated (35)	1.00	6.1	
	*DNMT3A*	Upregulated (11)	0.714	9	0.147
		Downregulated (36)	1.00	6	

(*) The number of patients who received the induction chemotherapy. Those were recategorized into 2 groups based on values of expression. The expression values above and below the 75th percentile were considered up and down-regulated, respectively.

**Table 5:** Association between each of the clinical laboratory data with overall and disease-free survival.

Variable name	Overall survival				Disease-free survival			
	*n*	Proportion surviving at 6 months	Median survival estimate (months)	*p*-value	*n*	Proportion surviving at 6 months	Median survival estimate (months)	*p*-value
All	49	0.598	18		47	0.56	6.33	
Age				0.869				0.553
Above median (40)	23	0.584	18		22	0.625	6.5	
Below median ( 40)	26	0.613	18		25	0.529	6.1	
Sex				0.865				0.977
Male	25	0.617	18		25	0.563	6	
Female	24	0.576	18		22	0.667	6.7	
*FLT3-*ITD	*n* = 45			< 0.001*	*n* = 43			< 0.0001*
Wild	39	0.662	18		37	0.714	6.5	
Mutant	6	NR	0.8		6	NR	0.2	
FAB subtypes	*n* = 48			0.107	*n* = 46			0.066
M1	12	0.367	1.5		12	0.4	6	
M2	9	0.778	18		9	0.6	9	
M3	13	0.831	NR		12	0.857	7	
M4 or more	14	0.55	NR		13	0.25	4.4	
Cytogenetics	*n* = 35		NR	0.59	*n* = 32			0.254
Normal	22	0.697	NR		23	0.625	6	
t(15;17)	13	0.875			9	0.8	8.2	

(*) indicates significance, NR means this group has not reached OS or DFS.

## Discussion

The present study assessed *CREBBP*,* CEBPA*, and *DNMT3A* expression in 53 adult AML patients from Egypt. Inconsistent with the literature, the present study detected the expression of *CEBPA* and *DNMT3A* in almost all samples, with frequencies of 88.7% and 96.2%, respectively^18,19,25,31^. On the other hand, *CREBBP* expression was below the detection level by quantitative RT-PCR in 43 (81.13%) out of 53 samples. Absent CREBBP was reported to be associated with AML in numerous human studies and knockout experiments on animal models^5,32–34^. Mice with homozygous *CREBBP* knockout showed an inherent proliferative defect in progenitor cells of the hematopoietic system during fetal development and died at embryonic day ten^34^. Heterozygous *CREBBP* ^+/−^ mice developed hematologic neoplasia after acquiring a somatic mutation in the *CREBBP* WT allele^33^.

We could not detect any significant differences between either of the three genes’ expressions based on sex or age at the time of diagnosis. Since *CREBBP* expression was not investigated in similar designs, our finding represents the literature for the first time. As for *CEBPA*, this is a confirmation of the finding by Krygeir et al. (2020)^19^ but not Gholami et al. (2019)^18^, who showed that *CEBPA* expression was significantly higher in males. In fact, their cases were restricted to familial AML with germline mutations in *CEBPA*, but unfortunately, *CEBPA* mutations in our study were not available. Our *DNMT3A* gene expression showed similar results to others regarding sex^23,24^. However, another study had shown that *DNMT3A* was significantly overexpressed in the younger age group; this finding was discordant with what was observed in our cohort^23^. This could be attributed to variations in ethnicity or mutations.

FAB classification of AML is based on the type and maturity of cells from which the leukemic clone has originated. The selected genes regulate hematopoietic cell development from early precursor stages until maturity. As mentioned, CREBBP balances hematopoietic stem cell lineages. A comparative evaluation of *CREBBP* expression levels across different stages of hematopoiesis has yet to be assessed^4^. For *CEBPA*, modulating expression level tunes the differentiation of early multipotential precursors. Mature granulocytes and monocytes showed lower *CEBPA* levels than hematopoietic progenitor cells^35^. More importantly, while upregulation of the *CEBPA* expression level boosts the granulocytic pathway, its downregulation is required for cells to progress toward the monocytic lineage^36^. Stem cells with double *DNMT3A*^−/−^ mutations showed a selective growth advantage, failure to switch from self-renewal to differentiation program, and increased expression of genes associated with self-renewal^22^. Analyzing the expression levels of the three genes studied among the various FAB subtypes revealed differences, albeit not statistically significant. The M3 subgroup showed no detectable *CREBBP*, and its *CEBPA* expression level was the lowest compared to other subtypes. This could be because *CREBBP* acts mainly on early progenitors and blast cells, while the clonal population in M3 is predominantly more mature promyelocytes, where its action is no longer needed. In contrast, *CEBPA* expression was reported to be significantly upregulated among this particular subgroup in many studies^19,31,36,37^, and the opposite has also been reported^38^. Our study may have limited power to detect this difference due to its small sample size.

Karyotyping data was available for 37 patients. Among these, 22 had normal karyotypes; thirteen were positive for PML-RARA fusion gene or t(15;17), one with t(8;21), and one with the unusually reported 47 XY + 19 karyotype. Our results show no significant differences in the expression level of either of the three genes in this regard. In contradiction with other exits, higher *CEBPA* expression was associated with abnormal karyotypes in the Iranian population but not in Egyptian or Polish ones^18,19,31^. Also, *DNMT3A* overexpression showed a highly significant association with a lower frequency of normal karyotype^23^. In this cohort, we identified a case with 47 XY + 19 karyotype and M4 subtype. The 32-year-old male patient had no detectable *CREBBP* expression. Trisomy 19 as a sole chromosomal aberration was reported in the literature; however, its clinical and prognostic impact requires further elucidation^39,40^.

In the present study a novel finding not detected elsewhere was that *CREBBP* was found to be the only gene significantly negatively associated with the total leukocytic count. However, Zhang et al. (2020) documented that *DNMT3A* expression was significantly higher with higher peripheral blood blasts (*p* = 0.006) but not with TLC^23^.

Another novel finding was the association of *DNMT3A* expression with the CD117 phenotypic marker. CD117 marker belongs to a highly diverse group of surface proteins expressed on many cell types. However, it was reported to be not associated with *DNMT3A* expression in work by Zhang et al. (2020), whereas 7/166 (4.2%) were positive for *c-KIT* mutation^23^, unlike our data, CD117 was positive in 19/52 (37%). However, there is a conflict regarding the association of CD117 with *CEBPA* mutations^11,15,35^. CD117, or c-KIT, is a proto-oncogene that encodes for a tyrosine kinase receptor responsible for the phosphorylation of multiple intracellular proteins via PI3K/AKT activation and p70S6K signaling^41^. Interestingly, it was found that AKT activation has a regulatory role in *DNMT3A* activity. *DNMT3A* dissociates with chromatin upon phosphorylation by AKT, resulting in hypomethylation at promoter CpG islands and increased gene expression^42^. The relation between both proteins is further confirmed by Dai et al. (2017), who conditionally knocked in *Dnmt3a* mutation in mice, and the result was an increase in CD117^+^ cells and expression of many p70S6K related proteins^43^. Furthermore, Celik et al. (2015) demonstrated that loss of *Dnmt3a* compromised long-term hematopoiesis, but the leukemic transformation was only initiated once the *c-Kit* mutation was introduced^44^. Herein, significantly higher *DNMT3A* expression was demonstrated in CD117-positive patients. This may be pointing at a role of CD117 in the transcription of *DNMT3A*, not only its activity, thus providing clinical support for the interrelationship retrieved from the animal experiments.

The correlation between the three genes’ expression levels was investigated to further clarify the relationship reported in the literature. Xu et al. (2013) studied the potential role of acetylation on *CEBPA* function by transfecting the human blood cell line K562 with a plasmid that expresses the *CREBBP* gene^30^. CEBPA repressed the gene’s transcription under investigation, and CREBBP reversed the effect exerted. Also, mutations in genes responsible for histone acetylation, including *CREBBP*, are associated with biallelic *CEBPA* mutations^11^. Moreover, the granulopoiesis function of CEBPA is known to be altered by the acetylation of its lysine residues^45^. Given this potential influence of CEBPA by CREBBP, we analyzed the correlation between both genes’ expression levels. A highly statistically significant correlation between their expression levels was found. This correlation could either be a direct functional interaction or a common downstream effect, a notion that needs further investigation.

To the best of our knowledge, no studies have conducted a correlation analysis of the expression level of these genes. Our findings may be shedding more light on the potential interaction between both genes in the complex network of AML pathogenesis. While DNMT3A is known to methylate CpG islands, modulating the transcription in promoters and enhancers of affected genes^22^, CREBBP was shown to be enriched in the same regions by ChIP-Seq analysis^41^. However, the negative correlation between *CEBPA* and *DNMT3A* showed a trend despite not being statistically significant. Lastly, our results did not detect any correlation between *CREBBP* and *DNMT3A* expression levels, contrary to the comptional Analysis.

The role of *CREBBP*,* CEBPA*, and *DNMT3A* in the outcome of AML is still an unsettled area of controversy. This study found no association between *CREBBP* regulation, OS, or DFS as defined. This result should be further confirmed or refuted in a larger cohort. Many authors showed similar results for *CEBPA *^37^; however, other researchers reported an association between higher *CEBPA* expression and better overall survival but not disease-free survival^31^. Krygier and colleagues analyzed the association of *CEBPA* expression with mortality in a cohort of 43 patients, yielding no significant association^19^, like ours. Regarding *DNMT3A*, only two reports have analyzed its expression level with survival in AML^23,24^. While one showed similar findings^20^, the other reported that higher *DNMT3A* expression was significantly associated with more prolonged overall and leukemia-free survival^22^. *FLT3*-ITD, or internal tandem duplication of the FMS-like tyrosine kinase 3 gene, is the most frequent among the *FLT3* mutations associated with AML^11^. *FLT3*-ITD, despite not being sufficient to induce AML, is associated with a higher relapse rate and poor survival^42^. In the present study *FLT3*-ITD mutation affects the patients’ OS significantly (*p* < 0.0001*). No significant coorelation was found between *FLT3*-ITD mutation and any of the expressoion of the studied genes. It is well known that AML is a very hetrogenous and OS is affected by many other factors, *FLT3*-ITD mutation effect on OS varies even among different other Egyptian cohorts^46^.

In the computational analysis, the correlations observed among *CEBPA*, *CREBBP*, and *DNMT3A* in AML samples highlight potential functional interplays. Although weak, the statistically significant correlations between *CEBPA*, *DNMT3A*, and *CREBBP* suggest that these gene interactions play a crucial role in the pathogenesis or progression of AML. However, the functional interplay between *CREBBP* and *CEBPA*, although non-significant in the computational Analysis, is more significant in the Egyptian cohort only. Whether this difference is cohort-specific or otherwise, it could reflect a different disease biology in Egyptian AML.

We lean towards the idea that Egyptian AML had a different disease biology. First, in another Egyptian study, *DNMT3A* gene expression was significantly higher among AML patients in relation to control^24^. Second, a loss of function (LOF) *DNMT3A* R882H mutation was also more prevalent in Egyptian adult AML 27%^24^ and 18%^28^. In fact, the expression level was higher in the mutated Egyptian samples, 55% vs. 40%, when compared to the wild-type AML; however, the difference did not reach the statistical level (*p* = 0.063)^24^. In a Chinese cohort, *DNMT3A* expression in the AML patients was found to be significantly higher than that of the ALL patients or normal controls (*p* = 0.002 or *p* < 0.001), but their mutational frequency was much lesser (6/57 (10.7%))^47^ than two other Egyptian cohorts (22/123 (17.9%) and 12/45(26.7%)). Moreover, Asfour et al. (2020) found a trend (*p* = 0.06) of increased *DNMT3A* expression in mutated *DNMT3A* group when compared to the wild type group^24,28^ while Chinese AML *DNMT3A* expression exert higher expression in the wild type group contarary to the Egyptian AML. In a Korean cohort, the frequency was 15.7%^25^. Lastly, in the American cohort, the frequency was 37/281(13%)^48^.

Third, several miRNAs were known to post-transcriptionally regulate *DNMT3A*. Overexpression of miR-143 decreased *DNMT3A* mRNA and protein expression^49^. But we found in Egyptian cohort that miRNA-143-5p to be among the top ten downregulated miRNAs (data in press). Also, among 50 adult non-M3 AML, miR-143 expression level was significantly decreased (*p* < 0.001) when compared to 50 healthy control, thus sparing the increased *DNMT3A* expression^50^. Similarly, 63 Chinese leukemia patients had a significantly lower relative miR-143 expression when compared with healthy controls (*p* = 0.004), and the expression levels of miR143 and *DNMTA3A* were negatively correlated (*r*=-0.663, *p* = 0.001). Overexpression of miR-143 decreased *DNMT3A* mRNA and protein expression^49^. In addition, another miRNA, miR-29a-3p, which proved complementarities to the 3-UTR of *DNMT3A* and *3B *^51^, exhibited a significant reduction in 90% of Egyptian AML patients^52^. Fourth, the reverse computational analysis conducted in this study was revealed. These data tip the balance towards a different disease biology in Egyptian adult AML patients.

## Conclusion

The correlations observed among *CEBPA*, *CREBBP*, and *DNMT3A* in AML samples highlight potential functional interplays. Although weak, the statistically significant correlations between *CEBPA* vs. *DNMT3A* and *CREBBP* vs. *DNMT3A* in the computational analysis suggest that these gene interactions play a crucial role in the pathogenesis or progression of AML. However, the functional interplay between *CREBBP* and *CEBPA*, although non-significant in the computational analysis, is stronger and in the opposite direction in the Egyptian cohort only. Whether this difference is cohort-specific or otherwise, it could reflect a different disease biology in Egyptian AML. These findings pave the way for further research into the molecular mechanisms of AML and hold promise for identifying novel targets for personalized therapeutic intervention or biomarkers for disease prognosis.

## Data Availability

The data sets used and analyzed during the current study are available from the Gharieb S. El-Sayyad (corresponding author) upon reasonable request”.
